# Metabolic network driven analysis of genome-wide transcription data from *Aspergillus nidulans*

**DOI:** 10.1186/gb-2006-7-11-r108

**Published:** 2006-11-15

**Authors:** Helga David, Gerald Hofmann, Ana Paula Oliveira, Hanne Jarmer, Jens Nielsen

**Affiliations:** 1 Fluxome Sciences A/S, Diplomvej, DK-2800 Kgs, Lyngby, Denmark; 2 Center for Microbial Biotechnology, BioCentrum-DTU, Technical University of Denmark, Søltofts Plads, DK-2800 Kgs, Lyngby, Denmark; 3 Center for Biological Sequence Analysis, BioCentrum-DTU, Technical University of Denmark, Kemitorvet, DK-2800 Kgs, Lyngby, Denmark

## Abstract

**Background:**

*Aspergillus nidulans *(the asexual form of *Emericella nidulans*) is a model organism for aspergilli, which are an important group of filamentous fungi that encompasses human and plant pathogens as well as industrial cell factories. Aspergilli have a highly diversified metabolism and, because of their medical, agricultural and biotechnological importance, it would be valuable to have an understanding of how their metabolism is regulated. We therefore conducted a genome-wide transcription analysis of *A. nidulans *grown on three different carbon sources (glucose, glycerol, and ethanol) with the objective of identifying global regulatory structures. Furthermore, we reconstructed the complete metabolic network of this organism, which resulted in linking 666 genes to metabolic functions, as well as assigning metabolic roles to 472 genes that were previously uncharacterized.

**Results:**

Through combination of the reconstructed metabolic network and the transcription data, we identified subnetwork structures that pointed to coordinated regulation of genes that are involved in many different parts of the metabolism. Thus, for a shift from glucose to ethanol, we identified coordinated regulation of the complete pathway for oxidation of ethanol, as well as upregulation of gluconeogenesis and downregulation of glycolysis and the pentose phosphate pathway. Furthermore, on change in carbon source from glucose to ethanol, the cells shift from using the pentose phosphate pathway as the major source of NADPH (nicotinamide adenine dinucleotide phosphatase, reduced form) for biosynthesis to use of the malic enzyme.

**Conclusion:**

Our analysis indicates that some of the genes are regulated by common transcription factors, making it possible to establish new putative links between known transcription factors and genes through clustering.

## Abstract 2

Genome-wide transcription analysis of *Aspergillus nidulans* grown on different carbon sources and a reconstruction of the complete metabolic network of this filamentous fungi are presented.

## Background

*Aspergillus *represents a large and important genus of filamentous fungi comprising human pathogens such as *A. fumigatus*, plant pathogens such as *A. flavus*, and important cell factories such as *A. niger, A. oryzae*, and *A. terreus*. Furthermore, *A. nidulans *has been extensively used as a model organism for eukaryotic cells. Despite their importance as human and plant pathogens and their extensive use in food, chemical, and pharmaceutical production, it was only recently that an initiative was undertaken to sequence the genomes of several *Aspergillus *spp. Thus, the genomes of three *Aspergillus *spp. have been published (*A. nidulans *[1], *A. oryzae *[2], and *A. fumigatus *[3]), and complete genomic sequencing of several other species has been finished or is ongoing. This has enabled analysis of the function of these important organisms at the genome level.

Aspergilli are natural scavengers and hence they have a very flexible metabolism that enables consumption of a wide range of carbon and nitrogen sources. Considering the high degree of flexibility in the metabolism of aspergilli, it is interesting to evaluate the function of the metabolic network in these organisms during growth on different carbon sources. We therefore undertook a study of the metabolism of *A. nidulans *at the genome level during growth on three different carbon sources: glucose, glycerol, and ethanol. These three carbon sources enter the central carbon metabolism at different locations, and they have been reported to result in widely different regulatory responses [4-8].

Our study involved genome-wide transcription analysis using *in situ *synthesized oligonucleotide arrays containing probes for 9,371 out of the 9,541 putative genes in the genome of *A. nidulans *[9]. In order to map the effects of carbon source on transcription, we used well controlled bioreactors to grow the cells. In recent years a few large-scale transcription studies have been conducted in *A. nidulans*, but so far none has covered the complete set of predicted genes in the genome. Sims and coworkers [10] used spotted DNA arrays to interrogate 2,080 open reading frames (ORFs) within the genome of *A. nidulans*, using as probes polymerase chain reaction (PCR) products from expressed sequence tags (ESTs), as well as gene sequences deposited in GenBank. The arrays were initially used in connection with an ethanol-to-glucose upshift batch experiment with a reference strain [10], and subsequently modified to study the effect of recombinant protein secretion on gene expression in *A. nidulans *by comparing the transcription profiles of a recombinant and a reference strain grown in chemostat cultures [11]. For other species of *Aspergillus*, a few studies on transcription profiling using microarray technology have been reported in the literature. These made use of spotted DNA arrays fabricated from EST sequences of selected genes (for example, *A. oryzae *[12], *A. flavus *[13-15], and *A. parasiticus *[15]) and other types of arrays (for example, for *A. terreus *[16]). Furthermore, studies similar to ours (aiming to map differences in gene expression during batch growth on different carbon sources, in particular glucose and ethanol) have been performed with other organisms, such as the filamentous fungi *A. oryzae *[12] and *Trichoderma reesei *[17], and the yeast *Saccharomyces cerevisiae *(many studies, with the first being that of DeRisi and coworkers [18]), with only the latter covering the complete genome.

In this work transcriptome data were analyzed using a recently developed consensus clustering algorithm [19]. Clustering of transcription data is valuable with respect to assigning function to genes, and this is particularly pertinent to *A. nidulans *because less than 10% of the 9,541 putative genes have been assigned a function (more than 90% of the 9,541 putative genes are called hypothetical or predicted proteins), based on automated gene prediction tools [9]. Using consensus clustering, we identified genes specifically relevant to the metabolism of the different carbon sources and, of particular, interest we identified nearly 200 genes that were significantly upregulated only during growth on glycerol versus growth on glucose and ethanol.

In order to study further the transcriptional response to growth on different carbon sources at the level of the metabolism, we used the transcription data to evaluate the operation of the metabolic network. For this purpose, we reconstructed the metabolic network of *A. nidulans *at the genome level, based on detailed metabolic reconstructions previously developed for *A. niger *[20], *S. cerevisiae *[21], and *Mus musculus *[22], as well as information on the genetics, biochemistry, and physiology of *A. nidulans*. The metabolic network reconstructed for *A. nidulans *contains 1,213 reactions and links 666 genes to metabolic functions. In the process of reconstruction, we assigned metabolic functions to 472 ORFs that had not previously been annotated, by employing tools of comparative genomics based on sequence similarity and using public databases of genes and proteins of established function. The metabolic reconstruction provided a framework for the analysis of transcriptome data. In particular, the metabolic network was used in combination with a recently developed algorithm [23] to identify global regulatory responses of the metabolism to variations in carbon source.

## Results

### Reconstruction of the metabolic network and ORF annotation

The metabolic network of *A. nidulans *was reconstructed using a pathway-driven approach, which resulted in the assignment of metabolic roles to 472 ORFs that had not previously been annotated (Table 1). The reconstructed metabolic network linked a total of 666 genes to metabolic functions, including 194 previously annotated ORFs in the *Aspergillus nidulans *Database [9]. The resulting network comprises 1,213 metabolic reactions, of which 1095 are biochemical transformations and 118 are transport processes (Table 1), as well as 732 metabolites. Out of the 1,213 reactions there are 794 that are unique (681 unique biochemical conversions and 113 unique transport processes), indicating that 419 of the reactions in the metabolic network are redundant. All the reactions in the metabolic network are listed in Additional data file 7 (Table S1), as are the abbreviations assigned to the metabolite names (Table S2). The reconstructed metabolic network is to our knowledge the largest microbial network reported to date [24].

### Transcriptional responses to changes in the carbon source

**Figure 1:**
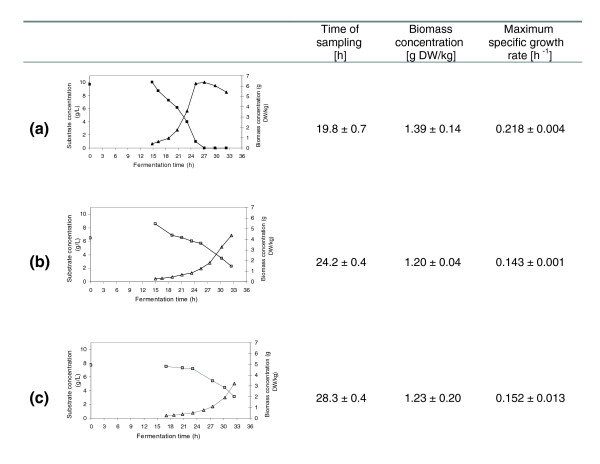
Biomass and substrate profiles for the different batch cultivations carried out with *A. nidulans*. **(a) **Cultivation with glucose as carbon source. **(b) **Cultivation with glycerol as carbon source. **(c) **Cultivation with ethanol as carbon source. For all cultivations, the time of sampling, the biomass concentration at the time of sampling, and the maximum specific growth rate for the culture are given.

In order to be able to identify primarily the effect of carbon source on transcription, we grew the cells in well controlled bioreactors, which enabled us to perform very reproducible fermentations. Figure 1 shows the biomass and substrate profiles for growth on glucose, glycerol, and ethanol. For the fermentations with glucose and glycerol as the carbon sources, the carbon recoveries were above 90% (>98% for glycerol), whereas it was only about 64% for growth on ethanol because of evaporation of the substrate. The batch fermentations were carried out in three replicates on each of the carbon sources investigated (for standard deviations, see Figure 1). For all of the cultivations, the samples for transcriptome analysis were taken in the early exponential phase of growth, with the biomass concentration being in the range of 1 to 1.5 g dry weight/kg. At this stage, dispersed filamentous growth was observed in all cultivations.

#### Identification of differentially expressed genes in pair-wise comparisons

The expression data for the three biological replicates on the three carbon sources were normalized (Additional data file 8 [Tables S3 to S5]) and compared in a pair-wise manner, in order to detect genome-wide transcriptional changes in response to a change in carbon source. Differentially expressed genes for each of the comparisons were identified by applying a significance statistical test (see Materials and methods, below) and considering a significance level (or cutoff in *P *value) of 0.01. Table 2 shows the total number of significantly regulated genes within the genome of *A. nidulans *for the three possible pair-wise comparisons between carbon sources, as well as the number of upregulated and downregulated genes. Because the change in carbon source is expected to result in changes in carbon metabolism, the number of differentially expressed genes that were comprised in the metabolic reconstruction for *A. nidulans *is also presented for each case. It is observed that there is an over-representation of metabolic genes that exhibit significant changes in expression (metabolic genes only comprise about 7% of the total number of genes). The complete list of genes whose expression was significantly changed in the pair-wise comparisons can be found in Additional data file 9 (Tables S6 to S8; they are also partly illustrated in Figures S1 to S3 in Additional data files 1, 2 and 3, respectively). The differentially expressed genes were functionally classified based on Gene Ontology (GO) assignments provided by CADRE [25] (Additional data file 10 [Tables S9 and S10]).

#### Gene clustering

**Figure 2:**
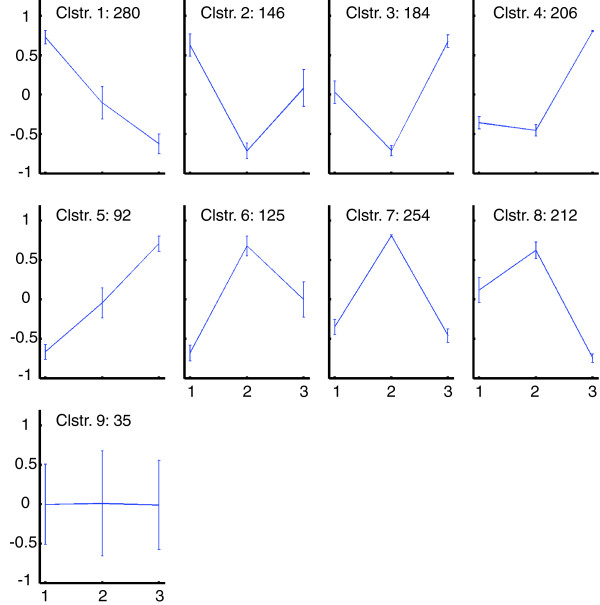
Representation of the eight clusters of genes identified. The numbers of genes in each cluster are as follows: 280 in cluster 1, 146 in cluster 2, 184 in cluster 3, 206 in cluster 4, 92 in cluster 5, 125 in cluster 6, 254 in cluster 7, and 212 in cluster 8. The x-axis represents the different carbon sources investigated: 1, glucose; 2, ethanol; and 3, glycerol. The y-axis represents normalized intensities, according to Grotkjær and coworkers [19]. Cluster 9 contains discarded genes, with low assignment to any of the other clusters.

The genes were arranged in clusters, according to their expression profiles. In order to reduce the noise in the expression data before clustering analysis, an analysis of variance (ANOVA) test was performed that considered normalized transcriptome data from all of the replicated experiments on the different carbon sources (Additional data file 11 [Table S11]). The complete list of statistically significant genes for different significance levels is presented in Additional data file 11 (Table S12). For a significance level (or cutoff in *P *value) of 0.05, it was observed that the expression levels of 1,534 genes were significantly changed, of which 251 represented metabolic genes. Clustering analysis was applied to these 1,534 genes, and a total of eight clusters were identified (along with an additional cluster that included discarded genes). These clusters are represented in Figure 2, and the genes belonging to each group are listed in Additional data file 12 (Table S13). The GO annotation available in CADRE [25] was also used for functional classification of the genes included in the different clusters (Table 3). The transcriptional patterns of these 1,534 differentially expressed genes were also used for hierarchical cluster analysis (data not shown), and it was observed that the replicated experiments clustered together, as expected.

#### Identification of metabolic subnetworks

In order to map overall metabolic responses to alterations of the carbon source, we applied the algorithm proposed by Patil and Nielsen [23] to identify the so-called reporter metabolites and to search for highly correlated metabolic subnetworks for each of the three pair-wise comparisons. This analysis relied on the reconstructed genome-scale metabolic network of *A. nidulans*, and hence we demonstrated how this metabolic network could be used to map global regulatory structures in *A. nidulans*. The top 15 high-scoring reporter metabolites for each of the cases are listed in Table 4 (also see Additional data files 4, 5 and 6 [Figures S4 to S6, respectively]).

**Figure 3:**
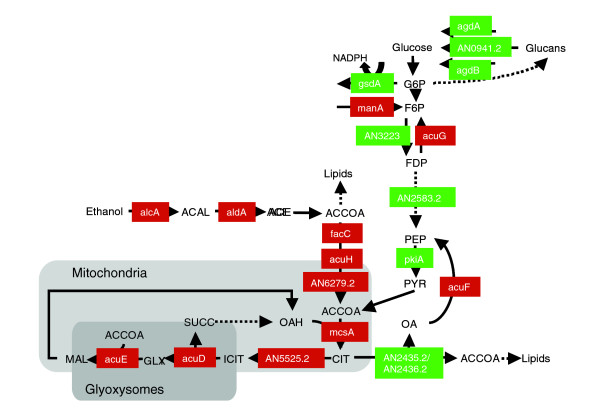
Small subnetwork identified for the shift from glucose to ethanol as carbon source. Genes marked red are upregulated and genes marked green are downregulated upon the shift. The metabolic map is simplified (many transport reactions are not included and the two steps of the glycoxylate pathway [encoded by the genes *acuD *and *acuE*] are placed in the mitochondria even though they are really located in the glyoxysomes). Conversions that involve several steps are indicated by dashed arrows. The metabolites are as follows: ACCOA, acetyl-CoA; ACE, acetate; ACHO, acetaldehyde; CIT, citrate; F16BP, fructose 1,6-bisphosphate; F6P, fructose 6-phosphate; G6P, glucose 6-phosphate; GLY, glyoxylate; ICIT, isocitrate; MAL, malate; OAA, oxaloacetate; PEP, phosphoenolpyruvate; PYR, pyruvate; SUC, succinate.

To identify metabolic subnetworks with co-regulated expression patterns we began by finding high-scoring subnetworks, using the whole reaction set in the reconstructed metabolic network for *A. nidulans*, and subsequently we repeated the algorithm to identify smaller subnetwork structures. The repetition of the algorithm resulted in more robust solutions and in the identification of smaller networks, as demonstrated earlier for yeast data [23]. Table 5 shows the list of enzymes and transporters comprising the 'small' subnetworks for each of the pair-wise comparisons between the three carbon sources investigated (also see Additional data files 4, 5 and 6 [Figures S4 to S6, respectively]). Figure 3 shows key enzymes and transporters comprising the 'small' subnetwork for the glucose versus ethanol comparison. The 'large' subnetworks are given in Additional data file 13 (Tables S14 to S16). The genes in each of the 'small' subnetworks were classified according to the GO-terms assigned, and the results are presented in Additional data file 14 (Table S17).

## Discussion

### Enzyme complexes

In the process of reconstructing the metabolic network we identified several multi-enzyme complexes (for example, the F_0_F_1 _ATP synthase complex or the pyruvate dehydrogenase complex, which consist of several different proteins), and we used the transcriptome data to assess whether there was coordinated control of the expression of genes encoding the proteins of these complexes. Thus, for each enzyme complex included in the metabolic reconstruction of *A. nidulans*, we investigated whether the corresponding subunits had similar expression profiles. This was checked by verifying whether the genes encoding proteins within each enzyme complex were assigned to the same clusters. Furthermore, we calculated the Pearson correlations for all possible combinations within each enzyme complex (data not shown), in order to evaluate how well the corresponding expression levels correlated to each other. Calculation of Pearson correlations also enabled analysis of genes whose expression did not change significantly in the conditions studied. Based on the clustering and Pearson correlation analyses, we observed that, for about 30% (8/27) of the enzyme complexes considered, the expression profiles of the genes encoding all of the subunits of each enzyme complex were similar. Furthermore, in 11% (3/27) of the cases, the transcription of at least 50% (and <100%) of the subunits within an enzyme complex were highly correlated.

We performed the same analyses for *S. cerevisiae *using transcription data for similar conditions [26]. Here we observed that for about 21% (4/19) of the enzyme complexes included in the metabolic model for yeast [21], all of the corresponding subunits had similar expression patterns. Moreover, for 11% (2/19) of the enzyme complexes there was high correlation for at least 50% (and <100%) of the genes encoding for the complexes. Despite co-regulation of enzyme complexes in both *A. nidulans *and yeast, there does not appear to be any conservation in terms of transcriptional regulation of enzyme complexes, because only 7% (2/27) of enzyme complexes in *A. nidulans *with co-regulation on different carbon sources (either all components or 50% of the components) were also found to be co-regulated in yeast.

### Ethanol utilization

The catabolism of ethanol, as well as regulation of the genes involved in this process, is presumably one of the best studied systems in *A. nidulans *(see Felenbok and coworkers [27] for a recent review). Two genes are responsible for the breakdown of ethanol into acetate via acetaldehyde, namely the genes encoding alcohol dehydrogenase I (*alcA*; AN8979.2) and aldehyde dehydrogenase (*aldA*; AN0554.2). The activation of this catabolic pathway is dependent on the transcriptional activator *alcR *(AN8978.2) [28]. Interestingly, a whole gene cluster composed of seven genes that are responsive to ethanol (or, more specifically, the gratuitous inducer methyl ethyl ketone) has previously been reported [29]. This cluster includes *alcA *and *alcR*, as well as five other transcripts (*alcP *[AN8977.2], *alcO*, *alcM *[AN8980.2], *alcS *[AN8981.2], and *alcU *[AN8982.2]), whose molecular functions have not yet been identified. In particular, one of these genes (*alcO*) has not been annotated in the genome sequence of *A. nidulans*, and similarity searches or gene prediction programs using the DNA sequence of the putative location of this gene were unsuccessful. Because our array design was based on annotated ORFs in the genome, this putative gene was not included in our analysis. However, all of the other genes of this cluster were found to be significantly upregulated on ethanol (*alcP*, *alcR*, *alcA*, *alcM*, and *alcS *were found in cluster 7, and *alcU *was found in cluster 6). Further positional analysis showed that there were no other gene clusters that were significantly regulated under any of the conditions studied (data not shown).

The subnetwork analysis clearly pointed to a coordinated expression of genes involved in ethanol metabolism upon shift from glucose to ethanol (Figure 3), and the response was to a large extent the same in the shift from glycerol to ethanol (Table 5). Ethanol is converted to acetate and is further catabolyzed to acetyl-coenzyme A (CoA), which then enters the mitochondria where it is oxidized (Figure 3). The subnetwork identified (Table 5) includes methylcitrate synthase (encoded by *mcsA*; AN6650.2), which was upregulated during growth on ethanol. This may point to a role of this enzyme in the catabolism of acetyl-CoA, in addition to the mitochondrial citrate synthase (encoded by *citA*; AN8275.2), which is expressed during growth both on glucose and ethanol. This is consistent with earlier reports in which it was found that this enzyme also possesses some citrate synthase activity [30].

The list of reporter metabolites (Table 4) is consistent with the identified subnetwork, because several components of the subnetwork are identified as reporter metabolites (CoA, acetyl-CoA, glyoxylate, oxaloacetate, carnitine, and *O*-acetyl-carnitine).

Besides *alcA *or ADH I (AN8979.2), *A. nidulans *has two additional alcohol dehydrogenases, namely *alcB *or ADH II (AN3741.2) and ADH III (AN2286.2). The former was assigned to cluster 6, whereas the latter did not appear to be significantly regulated in our analysis. It is interesting to observe that several genes in the identified subnetwork are also part of the metabolism of acetate, which is positively regulated by FacB (AN0689.2). Furthermore, *facB *was found to be significantly upregulated during growth on ethanol and assigned to cluster 7. FacB has been shown to induce directly the transcription of genes that are involved in the catabolism of acetate (acetyl-CoA synthetase, *facA *[AN5626.2]; carnitine acetyl transferase, *facC *[AN1059.2]; isocitrate lyase, *acuD *[AN5634.2]; malate synthase, *acuE *[AN6653.2]; and acetamidase, *amdS *[AN8777.2]) [5,6]. All of these genes were found to be significantly upregulated during growth on ethanol (assigned to cluster 7), and several of them are part of the subnetwork identified from the pair-wise comparison between glucose and ethanol (Table 5).

The subnetwork also included ATP:citrate oxaloacetate-lyase, which catalyzes the formation of acetyl-CoA and oxaloacetate from the reaction of citrate and CoA, with concomitant hydrolysis of ATP to AMP and phosphate. This enzyme represents a major source of cytosolic acetyl-CoA during growth on glucose, which is a precursor for lipid biosynthesis. In *A. nidulans*, ATP:citrate oxaloacetate-lyase appears to be regulated by the carbon source present in the medium, with high activity in glucose-grown cells and low activity in acetate-grown cells [31]. This may be due to the fact that, during growth on C2 carbon sources, acetyl-CoA is formed directly in the cytosol in connection with the catabolism of the carbon source. The genes encoding the enzyme complex for ATP:citrate oxaloacetate-lyase (AN2435.2 and AN2436.2) were among the most significantly downregulated genes upon shift from glucose to ethanol (decreases of 22.6-fold and 22.2-fold, respectively; Additional data file 9 [Table S6]). Moreover, the genes encoding ATP:citrate oxaloacetate-lyase fell into cluster 2, together with another group of genes that were downregulated upon a shift from glucose to ethanol, namely the major part of the enzymes in the pentose phosphate (PP) pathway (Additional data file 12 [Table S13]). The subnetwork also captured changes in the expression of genes participating in gluconeogenesis, glycolysis, and the PP pathway. It was observed that genes involved in gluconeogenesis (PEP carboxykinase and fructose 1,6-bisphosphatase) were upregulated during growth on ethanol (assigned to clusters 7 and 6, respectively), whereas many of the genes of the PP pathway were downregulated (assigned to cluster 2). This suggests that an energetically more favorable route for supply of NADPH (nicotinamide adenine dinucleotide phosphatase, reduced form) is used during growth on ethanol, namely through the malic enzyme (encoded by *maeA *[AN6168.2]), which was found to be upregulated during growth on ethanol and was identified in the subnetwork for the glycerol versus ethanol comparison. This is consistent with earlier findings that the activity of malic enzyme is low on glucose and high on ethanol [32], and that *maeA *may be weakly regulated by carbon catabolite repression [33].

From the above, it is clear that there is coordinated regulation of genes in very different parts of the metabolism, which is important for the cell to maintain homeostasis during growth on different carbon sources. The strength of our analysis based on the metabolic network is that these coordinated expression patterns are clearly captured using a nonsupervised algorithm.

For the ethanol versus glucose comparison, it was interesting to note that the gene with the greatest fold change (151 times) was that of *alcS*. This is relevant considering that no molecular function has been suggested for this gene so far. *In silico *analysis suggests that AlcS might be a membrane bound transporter protein (six transmembrane-helix domains; conserved domain [PFAM01184]), indicating that AlcS could be an acetate transporter.

### Regulation of transcription factors

As mentioned above, we observed that the gene *facB *was upregulated during growth on ethanol. However, we also found that several other transcription factors were regulated during growth on ethanol. Thus, we observed that *creA *(AN6195.2), which is the major mediator of carbon catabolite repression in *A. nidulans*, was located in cluster 6 and hence was upregulated during growth on ethanol. This might seem surprising, considering that CreA is assumed to be a transcriptional repressor and most active on glucose, but our findings corroborate findings reported by Strauss [34] and Sims [11] and their coworkers, who showed that *creA *is regulated at the transcriptional level when the mycelium is shifted to or from ethanol. The low expression of *creA *on glucose could be due to autoregulation, which is presumably elevated on the de-repressing carbon source ethanol, and on the intermediate repressing carbon source glycerol. However, our findings clearly showed that this regulation of *creA *not only occurs after changing the carbon source but is also reflected in the mRNA abundance of *creA*, during balanced growth conditions (it is not a transient phenomenon).

Besides the two transcriptional regulators AlcR and FacB, another known positive regulator was found in cluster 7, namely AreA (AN8667.2). AreA was probably the first regulatory gene described in *A. nidulans *[35], and it is a wide-domain regulator necessary for the activation of genes for the utilization of nitrogen sources. To our knowledge, it has not been reported that AreA is upregulated during growth on ethanol as compared with glucose or glycerol (cluster 7). Our results could indicate crosstalk between carbon repression and nitrogen repression pathways in *A. nidulans*. Supporting our findings on AreA regulation, we identified the gene *uapC *(AN6730.2) in cluster 7. This gene encodes a purine permease and has been shown to be regulated by AreA [36]. Another transcription factor assigned to cluster 7, namely *metR*, encodes a transcriptional activator for sulfur metabolism in *A. nidulans *[37], and it thereby links yet another branch of central metabolism to the regulatory network that is controlled by the nature of the carbon source.

### Glycerol utilization and polyol metabolism

Regulation of the biosynthesis and breakdown of glycerol are less studied in comparison with the metabolism of ethanol, but from our analysis we identified more than 200 genes that were significantly upregulated and another 200 genes that were significantly downregulated only during growth on glycerol as compared with growth on glucose and ethanol (clusters 4 and 8). It was previously described that there are two metabolic pathways that lead to glycerol, from the glycolytic intermediate dihydroxyacetone 3-phosphate. One of these pathways proceeds via dihydroxyacetone kinase to dihydroxyacetone, which is then converted into glycerol, by the action of a glycerol dehydrogenase (NADH [nicotinamide adenine dinucleotide] or NADPH dependent). The alternative route, which has been suggested to be responsible for the catabolism of glycerol [8], includes the formation of glycerol 3-phosphate (catalyzed by glycerol 3-phosphate dehydrogenase), and subsequently its conversion into glycerol, by the action of glycerol 3-phosphate phosphatase.

Several of the genes encoding these enzymes have previously been characterized, and we identified alternative candidates, as well as the missing ones, in our reconstruction of the metabolic network. The data obtained from the transcriptome analysis confirmed that the catabolic pathway via glycerol 3-phosphate is a major route for glycerol catabolism, because a gene putatively encoding the glycerol kinase (AN5589.2), as well as the gene putatively encoding a FADH-dependent glycerol 3-phosphate dehydrogenase (AN1396.2), were both significantly upregulated on glycerol as compared with ethanol and glucose. Moreover, both genes were assigned to cluster 4, which represents genes that are specifically upregulated during growth on glycerol, and were identified in the subnetworks of glycerol comparisons with the two other carbon sources. However, the transcriptome data also showed that the alternative pathway might be involved in the catabolism of glycerol. In fact, a gene that was identified in the metabolic reconstruction process as putatively encoding a NADPH-dependent glycerol dehydrogenase (AN7193.2) was upregulated on glycerol (cluster 3), as well as a gene that was identified as a putative dihydroxyacetone kinase (AN0034.2; cluster 4). Therefore, it seems likely that both pathways are actually involved in the utilization of glycerol. Interestingly, a previously characterized gene encoding a NADPH-dependent glycerol dehydrogenase (*gldB*; AN5563.2) [38] was also found to be significantly regulated, but exhibited a very different expression pattern from the putative gene encoding NADPH-dependent glycerol dehydrogenase (AN7193.2). Thus, because *gldB *was downregulated on glycerol, it was assigned to cluster 8.

The biosynthesis of mannitol occurs through routes that are similar to the two metabolic pathways that lead to glycerol. It has been reported that mannitol is implicated in the stress response to heat [39] and that it is the most abundant polyol in conidia of *A. nidulans *[40]. One of the pathways that lead to mannitol proceeds via mannitol 1-phosphate, from the glycolytic intermediate fructose 6-phosphate, and another one, which has fructose as an intermediate. The metabolic reactions interconverting these four metabolites open the possibility for a cyclic reaction pathway within the cell that allows the conversion of NADH into NADPH at the expense of one molecule of ATP [41]. None of the genes encoding enzymes involved in the mannitol cycle have previously been characterized. However, by applying the comparative genomics approach for the reconstruction of the metabolism, we identified putative candidate ORFs for all the reactions of the mannitol cycle, with the exception of the mannitol 1-phosphate phosphatase. Interestingly, most of these ORFs identified (6/8) showed lower expression levels on ethanol, at least when compared with glycerol (assigned to clusters 2, 3, and 4), and this could point to a role for the mannitol cycle in the formation of NADPH during growth on glycerol. Moreover, the gene that encodes the glucose 6-phosphate dehydrogenase (AN2981.2), which has been shown to be positively correlated with the formation of mannitol, was also assigned to cluster 2 and significantly downregulated on ethanol. This enzyme was identified in the subnetwork for the glucose versus glycerol comparison, and transcription of the corresponding gene was lower during growth on glycerol than during growth on glucose. This could indicate a partial shift from the PP pathway, as the main route for NADPH supply for biosynthesis, to the mannitol cycle.

Glycerol has also been shown to be involved in the response to different osmotic conditions in *A. nidulans *[42], and it has also been reported that all of the components of the high-osmolarity glycerol (HOG) response pathway that are known in yeast have orthologs in *A. nidulans *[43,44]. The analysis of the transcriptional responses of these components to the different growth conditions considered in the present study revealed that only the gene that encodes the sensor protein SlnA (*slnA*; AN1800.2) was significantly regulated and this was assigned to cluster 4 (*slnA *seemed to be induced when glycerol was the sole carbon source, as compared with glucose or ethanol).

### Metabolism of reserve compounds and cell wall polysaccharides

Another metabolite that has been reported to be related to glycerol metabolism is trehalose. In fact, it has been shown that trehalose, which is stored in the conidiospores, is converted into glycerol upon germination [45].

The biosynthesis of trehalose occurs, via trehalose 6-phosphate, from glucose 6-phosphate and UDP-glucose, whereas it is degraded directly to glucose. Our reconstruction of the metabolic network includes six genes that might be involved in these metabolic pathways, of which four have been confirmed experimentally [45-48]. The cluster analysis showed that the transcription of three of these six genes was significantly changed, with higher levels on glucose, compared with ethanol and glycerol (genes assigned to clusters 1 and 2). Because these three genes encode each of the different steps in the biosynthesis as well as degradation of trehalose, these observations suggest that there may be a higher turnover of trehalose during growth on glucose.

Glycogen is another reserve carbohydrate, similar to trehalose, and interestingly the two genes putatively assigned to its biosynthesis and degradation exhibited their highest expression levels on glycerol (clusters 3 and 4, respectively), which might suggest an effect of this carbon source on glycogen turnover. In this regard, it was also interesting to verify that the GO term analysis for the pair-wise comparisons showed that genes associated with cell wall metabolism were significantly over-represented in the upregulated gene set as well as in the downregulated gene set.

More detailed analysis of the genes that were upregulated on glycerol compared with glucose, and that resulted in the over-representation of the GO terms, revealed that all of them putatively encode enzymes with β-1,3-glucosidase activity, which suggests that specially the β-1,3-glucan fraction of the fungal cell wall undergoes major rearrangements depending on the carbon source. On the other hand, the genes that were downregulated on glycerol and associated with GO terms for the cell wall biosynthesis encoded α-glucosidases (AN8953.2, AN0941.2, and AN4843.2) and were assigned to cluster 5. These enzymes are responsible for the breakdown of α-linked glucans into glucose, and it is therefore surprising that three genes encoding α-glucosidases (one putatively [AN0941.2] and two experimentally confirmed [*agdA *(AN2017.2) and *agdB *(AN8953.2)] [49]) exhibited their highest expression levels on glucose, which means that they are not repressed by glucose. It could be speculated that these genes are also involved in the remodeling of the α-glucan fraction of the cell wall, depending on the available carbon source.

One of the α-glucosidases (AN2017.2) is part of a gene cluster that encodes proteins responsible for the breakdown of α-glucans (such as starch). This cluster contains a putative glycosyl transferase (AN2015.2) that was significantly downregulated on ethanol compared with glucose and glycerol (assigned to cluster 3); the previously mentioned *agdA*, which encodes an α-glucosidase; the regulatory protein *amyR *(AN2016.2), which appears to be regulated in the same way as *agdA *(also found in cluster 2 and significantly downregulated on ethanol compared with glucose); and, finally, *amyA *(AN2018.2), which encodes an α-amylase but which does not appear to be significantly regulated under the conditions investigated in the present study.

AmyR directly controls the expression of *agdA *by binding to its promoter [50] and the direct correlation between the two mRNA levels suggests that solely the quantity of AmyR within the cell might be responsible for the regulation of *agdA*, without any further requirement for post-translational activation of AmyR. It is also interesting to note that it has previously been shown that *amyR *is controlled by CreA [51], which is in agreement with our findings (compare the expression pattern of cluster 6, containing *creA*, with that of cluster 2, containing *amyR*).

### Ribosome biogenesis

It has been reported that the specific growth rate influences the expression of genes encoding ribosomal proteins in *S. cerevisiae*, and that the transcription of these genes increases with increasing specific growth rates [52]. Similarly, we observed that the expression profiles of genes encoding ribosomal proteins followed the same trend as the maximal specific growth rate. In fact, in the batch cultivations carried out with *A. nidulans *on the different carbon sources the cells grew at unlimited conditions, and hence at their maximal specific growth rate possible on the given carbon source. The specific growth rates were highest for growth on glucose and about the same on ethanol and glycerol (Figure 1). According to the GO term analysis, cluster 1 is mainly characterized by genes whose functions are related to ribosome biogenesis, as can be observed in Table 3. Indeed, 59 out of the 280 genes assigned to cluster 1 are associated with the GO term 'ribosome biogenesis'. It is interesting that this cluster includes genes that have higher expression levels during growth on glucose, and this indicates - as observed for yeast - that these genes might indeed be involved in the ribosome biogenesis and that they are possibly regulated in a growth-rate dependent manner.

## Materials and methods

### Strain

The strain used in this study was the strain *Aspergillus nidulans *A187 (pabaA1 yA2; obtained from the Fungal Genetics Stock Center, Kansas City, KA, USA).

### Growth medium

The medium used in all batch cultivations was a chemically defined medium as described by Agger and coworkers [53], with the following modifications: NH_4_Cl was used as the nitrogen source, at a concentration of 12.2 g/l, and three different carbon sources were tested, namely glucose, glycerol, and ethanol (10 g/l). Yeast extract was added to the fermenter at a concentration of 3 mg/l in order to encourage the germination of spores. Furthermore, the nutritional supplement *p*-aminobenzoic acid (PABA) was added to the medium at a concentration of 1 mg/l, as well as the antifoam agent 204 (Sigma, Brøndby, Denmark)) at a concentration of 0.05 ml/l.

### Propagation of spores

The fermenters were inoculated with spores of *A. nidulans *A187 previously propagated on solid minimal medium [54] containing PABA (10 mg/l). The same stock of spores of *A. nidulans *was used to inoculate all the plates. The spores were cultivated at 37°C, during 3 to 4 days, and harvested by adding distilled water. For the fermentations performed in replicates, the fermenters were inoculated with the same solution of spores, to a final concentration of 5 × 10^9 ^spores/l. High concentrations of spores in the inoculum were employed because they favor dispersed filamentous growth. The spore solutions were vortex mixed before introduction into the fermenters, in order to prevent agglomeration of spores and thus pellet formation.

### Batch cultivations

All aerobic batch cultivations were performed in 3 L-Braun fermenters with a working volume of 2 l. The bioreactors were equipped with two Rushton four-blade disc turbines and no baffles (thereby reducing the surface available for wall growth). Air was used to sparge the bioreactor. The concentrations of oxygen and carbon dioxide in the exhaust gas were monitored using an acoustic gas analyzer (Brüel & Kjær, Nærum, Denmark). Temperature, pH, agitation, and aeration rate were controlled throughout the cultivations. The temperature was maintained at 30°C. The pH was controlled by automatic addition of 2 N NaOH and 2 N HCl. For the cultivations on glucose and glycerol, the pH was initially set to 3.0 to prevent spore aggregation; only when the spores started to germinate was the pH gradually increased to 6.0. Because pellet formation is unlikely to occur during growth on ethanol, the pH was set to 6.0 from the start, in the cultivations performed with this carbon source. Similarly, the stirrer speed was initially set to 100 rpm and the aeration rate to 0.01 vvm, and only after the spores started to germinate were these parameters progressively increased to 700 rpm and 1 vvm, respectively.

### Sampling

For quantification of cell mass and extracellular metabolites, the fermentation broth was withdrawn from the fermenter vessel and filtered through nitrocellulose filters (pore size 0.45 μm; Pall Corporation, East Hills, NY, USA). The filter cakes were immediately processed for determination of cell mass, whereas the filtrates were stored at -20°C until they were analyzed for determination of extracellular metabolites (substrates and metabolic byproducts).

For gene expression analysis, the mycelia were harvested and processed within half a minute. The mycelia were filtered using Miracloth (Calbiochem, San Diego, CA, USA) and washed with phosphate-buffered saline (PBS) solution (137 mmol/l NaCl, 2.7 mmol/l KCl, 10 mmol/l Na_2_HPO_4_, and 0.24 mmol/l KH_2_PO_4_; pH 7.4). After removal of excess PBS solution, the mycelia were frozen in liquid nitrogen and stored at -80°C until further analysis.

### Cell mass determination

Cell dry weight was determined using nitrocellulose filters (pore size 0.45 μm; Pall Corporation). The filters were predried in a microwave oven at 150 W for 10 minutes and subsequently weighed. A known volume of cell culture was filtered and the filter cake was washed with 0.9% NaCl and dried on the filter for 15 minutes in a microwave oven at 150 W. The filter was weighed again to determine the cell mass concentration.

### Analysis of extracellular metabolites

The concentrations of sugars, organic acids, and polyols in the filtrates (thawed on ice) were determined using high-pressure liquid-chromatography on an Aminex HPX-87Hm column (Bio-Rad, Hercules, CA, USA). The column was kept at 65°C and eluted at 0.6 ml/minute with 5 mmol/l H_2_SO_4_. The compounds were detected refractometrically (410 Differential Refractometer, Waters Corporation, Milford, MA, USA).

### Extraction of total RNA

Total RNA was isolated using the Qiagen RNeasy Mini Kit (QIAGEN Nordic, Ballerup, Denmark), according to the protocol for isolation of total RNA from animal tissues. For this purpose, approximately 20 to 30 mg frozen mycelium was placed in a 2 ml Eppendorf tube, precooled in liquid nitrogen, containing three RNase-treated steel balls (two balls with a diameter of 2 mm and one ball with a diameter of 5 mm). The tubes were then shaken in a Retsch Mixer Mill, at 3°C, for 6 to 8 minutes, until the mycelia were ground to powder and thus ready for extraction of total RNA. The quality and quantity of the total RNA extracted were determined by spectrophotometric analysis and by gel electrophoresis. The total RNA was stored at -80°C until further processing.

### Microarray manufacturing and design

The microarrays used for the analysis of the transcriptome of *A. nidulans *were custom-made NimbleExpress™ arrays (NimbleGen Systems Inc., Madison, WI, USA), which were acquired through Affymetrix (Santa Clara, CA, USA). NimbleExpress™ arrays are manufactured using a Maskless Array Synthesizer system, which makes use of a Digital Micromirror Device. This device consists of a system of miniature aluminum mirrors, which are individually controlled, enabling the synthesis of oligomers in a similar way to the photolithographic process used in the manufacture of Affymetrix^® ^GeneChip arrays. The NimbleExpress™ arrays are packaged in an Affymetrix^® ^GeneChip cartridge (49 format), and can be used with GeneChip reagents and processed on the GeneChip^® ^Instrument System (for more information, see [55]).

The selection of the probes for interrogating the ORFs within the genome of *A. nidulans *was performed by the Affymetrix Chip Design Group, who used the same algorithms employed in the design of standard Affymetrix^® ^GeneChip arrays. The arrays contain only perfect match (PM) probes. Of the 9,541 putative genes identified in the genome of *A. nidulans *(*Aspergillus nidulans *Database [9], release 3.1; Broad Institute), 9,444 were represented in the microarray (CBS01CMBA530008N). Each ORF was interrogated with one (8,628), two (815), or three probe sets (1), and each of these probe sets were composed of 11 probes (whenever possible) of 25 oligomers. Different types of probe sets were represented on the array, namely type 1 (if all probes in the set hybridized exclusively with the target sequence), type 2 (if all probes in the set hybridized with the target sequence and cross-hybridized with other sequences), and type 3 (mixed probe set). In the data analysis, only probe sets of type 1 (or, rather, probes that did not cross-hybridize with genes other than the target) were considered, which brought the number of putative genes investigated down to 9,371.

### Preparation of biotin-labeled cRNA and microarray processing

Biotin-labeled cRNA was prepared from approximately 10 μg of total RNA, according to the protocol described in the Affymetrix GeneChip^® ^Expression Analysis Technical Manual (2004) [56]. An additional cleanup step was performed before fragmentation, using the Qiagen RNeasy Mini Kit (protocol for RNA Cleanup), in order to guarantee good-quality cRNA samples for subsequent processing. The cRNA was quantified in a spectrophotometer (Amersham Pharmacia Biotech, GE Healthcare Bio-Sciences AB, Uppsala, Sweden) and its quality was assessed by gel electrophoresis and using a bioanalyzer (Agilent Technologies Inc., Santa Clara, CA, USA). The biotin-labeled cRNA was then fragmented and approximately 13 μg of fragmented cRNA was hybridized to the custom-made NimbleExpress™ array (CBS01CMBA530008N), as described in the Affymetrix GeneChip^® ^Expression Analysis Technical Manual (2004) [56]. The array was further processed on a GeneChip^® ^Fluidics Station FS-400 (fluidics protocol EukGE-WS2v4) and on an Agilent GeneArray^® ^Scanner. The scanned probe array images (.DAT files) were converted into .CEL files using the GeneChip^® ^Operating Software (Affymetrix).

### Reconstruction of the metabolic network and gene annotation

The metabolic network of *A. nidulans *was reconstructed by employing a pathway-driven approach described elsewhere [57] and comparative genomics tools based on sequence similarity. The metabolism of *A. nidulans *was reconstructed using as templates detailed metabolic reconstructions previously developed for *A. niger *[20], *Saccharomyces cerevisiae *[21], and *Mus musculus *[22]. Pathway prediction in *A. nidulans *was supported by available information on its genetics, biochemistry, and physiology. Thereby, it was possible to identify metabolic functions without a gene associated in the genome of *A. nidulans*, and thus candidate ORFs for encoding those functions, by employing similarity-based tools of comparative genomic analysis (BLAST [Basic Local Alignment Search Tool]) and using public (nonredundant) databases of genes and proteins of established function [58].

### Analysis of transcriptome data

Raw probe intensities were preprocessed by applying the RMA (robust multiarray average) method for background correction (based on PM probe information) [59], and were subsequently normalized using the qspline method [60]. Gene expression index values were calculated using the method developed by Li and Wong [61], using the PM-only model. Normalized gene expression data was deposited at the GEO database [62], with accession numbers GPL3604 (platform), GSM102371-GSM102379 (samples), and GSE4578 (series).

Statistical analysis was applied to determine differentially expressed genes between the categories of replicated experiments. For all of the possible pair-wise comparisons within the three different categories, the logit-t method [63] was employed, whereas ANOVA was used to compare gene expression levels among all categories. The genes whose expression was found to be significantly changed using the ANOVA test were further arranged into clusters, by applying ClusterLustre, a Bayesian consensus clustering method recently developed and available in the Matlab application [19].

Furthermore, reporter metabolites (metabolites around which the most significant changes in transcription occur) and highly correlated metabolic subnetworks (sets of connected genes with significant and coordinated transcriptional response to a perturbation) were identified for each of the pair-wise comparisons possible within the three categories, as described by Patil and Nielsen [23]. For this purpose, information on the topology of the reconstructed metabolic network of *A. nidulans *was used in combination with the *P *values from the logit-t test performed for each of the pair-wise comparisons of expression data.

### Data deposition

Normalized gene expression data were deposited at the GEO database [62], with accession numbers GPL3604 (platform), GSM102371-GSM102379 (samples), and GSE4578 (series).

## Additional data files

The following additional data are available with the online version of this paper. Additional data file 1 illustrates genes whose expression was significantly changed in the pair-wise comparison: glucose versus ethanol. Additional data file 2 illustrates genes whose expression was significantly changed in the pair-wise comparison: glycerol versus ethanol. Additional data file 3 illustrates genes whose expression was significantly changed in the pair-wise comparisons glucose versus glycerol. Additional data file 4 illustrates reporter metabolites and enzymes comprising the 'small' subnetwork identified by comparing expression data on glucose and ethanol. Additional data file 5 illustrates reporter metabolites and enzymes comprising the 'small' subnetwork identified by comparing expression data on glycerol and ethanol. Additional data file 6 illustrates reporter metabolites and enzymes comprising the 'small' subnetwork identified by comparing expression data on glucose and glycerol. Additional data file 7 lists all of the reactions in the metabolic network reconstructed for *A. nidulans *and gives the abbreviations assigned to the metabolite names. Additional data file 8 lists the normalized intensities for the three biologic replicates on the three carbon sources studied. Additional data file 9 includes complete lists of genes whose expression was significantly changed in the pair-wise comparisons. Additional data file 10 presents the functional classification of the differentially expressed genes based on GO assignments provided by CADRE. Additional data file 11 provides results from the ANOVA test performed, considering normalized transcriptome data from all of the replicated experiments on the different carbon sources. Additional data file 12 lists the genes assigned to each gene cluster. Additional data file 13 presents the 'large' subnetworks for each of the pair-wise comparisons between the three carbon sources investigated. Additional data file 14 presents the functional classification of the genes in each of the 'small' subnetworks, based on GO assignments provided by CADRE.

## Supplementary Material

Additional data file 1 Differentially expressed genes in the central metabolism of *A. nidulans *between the replicated experiments on glucose and ethanol, revealed by the logit-t method (see Additional data file 9 [Table S6] for a full list of genes. Upregulated and downregulated genes are represented in red and green, respectively. Fold changes greater than 10 are highlighted in bold. Click here for file

Additional data file 2 Differentially expressed genes in the central metabolism of *A. nidulans *between the replicated experiments on glycerol and ethanol, revealed by the logit-t method (see Additional data file 9 [Table S7] for a full list of genes. Upregulated and downregulated genes are represented in red and green, respectively. Fold changes greater than 10 are highlighted in bold. Click here for file

Additional data file 3 Differentially expressed genes in the central metabolism of *A. nidulans *between the replicated experiments on glucose and glycerol, revealed by the logit-t method (see Additional data file 9 [Table S8] for a full list of genes. Upregulated and downregulated genes are represented in red and green, respectively. Fold changes greater than 10 are highlighted in bold. Click here for file

Additional data file 4 Reporter metabolites and enzymes comprising the 'small' subnetwork identified by comparing expression data on glucose and ethanol (represented in blue). Also shown are the top 15 high-scoring reporter metabolites. Click here for file

Additional data file 5 Reporter metabolites and enzymes comprising the 'small' subnetwork identified by comparing expression data on glycerol and ethanol (represented in blue). Also shown are the top 15 high-scoring reporter metabolites. Click here for file

Additional data file 6 Reporter metabolites and enzymes comprising the 'small' subnetwork identified by comparing expression data on glucose and glycerol (represented in blue). Also shown are the top 15 high-scoring reporter metabolites. Click here for file

Additional data file 7 Table S1 lists the reactions comprising the metabolic reconstruction developed for *A. nidulans*. Each reaction is associated to an ORF within the genome of *A. nidulans *(when available), and to the EC number of the corresponding enzyme (when available). Table S2 lists metabolites, abbreviations used, and full names. (In the abbreviations, the suffixes *m*, *g*, and *e *stand for metabolites localized in the mitochondria, glyoxysomes and extracellular medium, respectively. No suffix was added to cytosolic metabolites.) Click here for file

Additional data file 8 Tables S3, S4, and S5 give normalized intensities considering the replicated experiments on glucose and ethanol, on glycerol and ethanol, and on glucose and glycerol, respectively. Click here for file

Additional data file 9 Tables S6, S7 and S8 list differentially expressed genes between the replicated experiments on glucose and ethanol, revealed by the logit-t method. The genes are sorted according to ascending *P *value from the statistical analysis. The average fold changes of expression between ethanol and glucose (Table S6), between ethanol and glycerol (Table S7), and between glycerol and glucose (Table S8) are also presented (a fold change equal to 2 [-2] indicates a twofold upregulation [downregulation] in ethanol relative to glucose [Table S6], in ethanol relative to glycerol [Table S7], or in glycerol relative to glucose [Table S8]). Click here for file

Additional data file 10 Tables S9 and S10 present the classifications of the upregulated and downregulated genes, respectively, given in Table 2 into GO categories (provided by CADRE), according to the three most important biological processes and molecular functions. Click here for file

Additional data file 11 Table S11 gives the normalized intensities considering all of the categories of replicated experiments (glucose, glycerol, and ethanol). Table S12 provides *P *values from the ANOVA test between all categories of replicated experiments (glucose, glycerol, and ethanol). The genes are sorted according to ascending *P *value. Click here for file

Additional data file 12 Table S13 summarizes gene clusters identified by applying ClusterLustre to the genes that were found to be significantly changed in the ANOVA test. Click here for file

Additional data file 13 Tables S14, S15, and S16 provide lists of enzymes and transporters comprising the 'large' subnetwork, obtained by comparing expression data on glucose and ethanol, on glycerol and ethanol, and on glucose and glycerol, respectively. Click here for file

Additional data file 14 Table S17 summarizes the classification of the genes included in the 'small' highly correlated subnetworks into GO categories (provided by CADRE), according to the three most important biological processes and molecular functions. Click here for file

## Figures and Tables

**Table 1:** Biochemical conversions and transport processes, and number of ORFs associated with the metabolic reactions

Part of metabolism	Number of metabolic reactions	Number of previously annotated ORFs^a^	Number of newly annotated ORFs	Total number of ORFs
Biochemical reactions	1,095 (681^b^)	188	468	656
C-compound metabolism	463 (220)	96	166	262
Energy metabolism	20 (17)	14	40	54
Aminoacid metabolism	238 (171)	40	125	165
Nucleotide metabolism	144 (114)	10	44	54
Lipid metabolism	175 (122)	13	97	110
Secondary metabolism	42 (25)	16	14	30
Nitrogen and sulphur metabolism	8 (7)	2	3	5
Polymerization, assembly and maintenance	5 (5)			

Transport processes	118 (113)	6	3	9

Total	1,213 (794)	194	472	666

Shown are the total number of biochemical conversions and transport processes included in the metabolic reconstruction for *A. nidulans *(number of unique reactions are given in parenthesis), and the number of ORFs (previously and newly annotated) associated with the metabolic reactions. The total number of unique ORFs in the metabolic network may be different from the sum of the number of ORFs in the different parts of the metabolism, because there are ORFs that encode functions in several parts of the metabolism. ^a^*Aspergillus nidulans *Database [9]. ^b^Six nonenzymatic steps are included. ORF, open reading frame.

**Table 2:** Genes that are differentially expressed in the different pair-wise comparisons possible between the categories

Comparison	Total genes (up/down)	Metabolic genes (%)
Ethanol versus glucose	418 (249/169)	103 (25%)
Ethanol versus glycerol	206 (92/114)	58 (28%)
Glycerol versus glucose	71 (57/14)	12 (17%)

Shown are the number of genes that are differentially expressed in the different pair-wise comparisons possible between the categories, for a cutoff *P *value in the logit-t test of 0.01. The total number of genes is presented along with the number of upregulated (up) and downregulated (down) genes (shown in parenthesis). The number (and percentage) of metabolic genes identified within the differentially expressed genes is also shown.

**Table 3:** Classification of the genes in each cluster into GO categories

Cluster	Number of genes in cluster	Biological processes	Molecular functions
Cluster 1	280	Ribosome biogenesis Cytoplasm organization and biogenesis Ribosome biogenesis and assembly	RNA binding SnoRNA binding Nucleic acid binding
Cluster 2	146	Alcohol metabolism Monosaccharide metabolism Monosaccharide catabolism	Translation elongation factor activity Carbohydrate kinase activity Thryptophan synthase activity
Cluster 3	184	Karyogamy Karyogamy during conjugation with cellular fusion Glucan metabolism	DNA binding Protein kinase regulator activity Kinase regulator activity
Cluster 4	206		Peroxidase activity Oxidoreductase activity, acting on peroxide as acceptor
Cluster 5	92		Oxidoreductase activity Pyruvate dehydrogenase activity Pyruvate dehydrogenase (acetyl transferring) activity
Cluster 6	125	Generation of precursor metabolites and energy Energy derivation by oxidation of organic compounds Fatty acid β-oxidation	Oxidoreductase activity Triose-phosphate isomerase activity Allophanate hydrolase activity
Cluster 7	254	Cofactor metabolism Coenzyme metabolism Generation of precursor metabolites and energy	Hydrogen ion transporter activity Monovalent inorganic cation transporter activity Lyase activity
Cluster 8	212	Protein biosynthesis Cellular biosynthesis Macromolecule biosynthesis	Structural constituent of ribosome Structural molecule activity Peptidyltransferase activity

The genes in each cluster are classified into GO categories (provided by CADRE), according to the three most important biological processes and molecular functions. The fields with fewer than three categories correspond to cases in which the *P *values were above the cutoff selected in the GO term analysis. The sum of the number of genes in each cluster is not equal to the total number of differentially expressed genes (1,534) because 35 genes were discarded in the clustering analysis (see Analysis of transcriptome data, under Materials and methods).

**Table 4:** Highly regulated or reporter metabolites for the three possible pair-wise comparisons between the different carbon sources

Ethanol versus glucose			Ethanol versus glycerol			Glycerol versus glucose		
Reporter metabolite	*n*	*P*	Reporter metabolite	*n*	*P*	Reporter metabolite	*n*	*P*

Acetyl coenzyme A (mitochondrial)	12	2.1E-06	Oxaloacetate	13	7.6E-05	*N*-Carbamoyl-L-aspartate	3	1.0E-03
Coenzyme A (mitochondrial)	14	2.6E-06	Coenzyme A (mitochondrial)	14	1.2E-04	Carbamoyl phosphate	5	1.7E-03
Glyoxylate (glyoxysomal)	3	1.8E-05	Glyoxylate (glyoxysomal)	3	2.1E-04	2-(Formamido)-N1-(5'-phosphoribosyl)acetamidine	2	2.8E-03
Oxaloacetate	13	9.4E-05	Acetyl coenzyme A (mitochondrial)	12	2.7E-04	Glycogen	2	2.8E-03
Acetyl coenzyme A (glyoxysomal)	2	1.1E-04	Acetyl coenzyme A (glyoxysomal)	2	4.2E-04	Maltose	6	2.9E-03
Coenzyme A (glyoxysomal)	2	1.1E-04	Coenzyme A (glyoxysomal)	2	4.2E-04	Maltose (extracellular)	6	2.9E-03
Oxaloacetate (mitochondrial)	11	4.4E-04	Oxaloacetate (mitochondrial)	11	4.3E-04	L-glutamine	16	3.1E-03
Carnitine	2	4.9E-04	2-Oxoglutarate (mitochondrial)	9	4.9E-04	α-D-glucose 1-phosphate	4	3.4E-03
O-acetylcarnitine	2	4.9E-04	Citrate	1	5.6E-04	ATP	94	3.7E-03
Propanoyl-coenzyme A	3	6.1E-04	Phosphoenolpyruvate	6	8.5E-04	(R)-3-Hydroxy-3-methyl-2-oxobutanoate (mitochondrial)	2	4.4E-03
Maltose	6	7.0E-04	Fumarate (mitochondrial)	3	8.6E-04	(R)-2,3-dihydroxy-3-methylbutanoate (mitochondrial)	2	4.4E-03
Maltose (extracellular)	6	7.0E-04	α-D-glucose 1-phosphate	4	9.5E-04	Carbon dioxide	42	4.7E-03
O-acetylcarnitine (mitochondrial)	2	9.0E-04	Citrate (mitochondrial)	5	1.3E-03	S-acetyldihydrolipoamide (mitochondrial)	2	5.1E-03
Carnitine (mitochondrial)	2	9.0E-04	Carnitine	2	1.9E-03	Carbon dioxide (mitochondrial)	16	6.0E-03
O-acetylcarnitine (glyoxysomal)	2	9.0E-04	O-acetylcarnitine	2	1.9E-03	ADP	64	1.2E-02

Shown are highly regulated or reporter metabolites for the three possible pair-wise comparisons between the different carbon sources, according to Patil and Nielsen [23]. '*n*' denotes the number of neighbors of the reporter metabolite (the number of reactions in which it participates).

**Table 5:** Enzymes and transporters in subnetworks

Ethanol versus glucose (26 reactions)	Ethanol versus glycerol (33 reactions)	Glycerol versus glucose (34 reactions)
6-Phosphofructokinase	1,3-β-Glucan synthase	5'-Phosphoribosylformyl glycinamidine synthetase
Acetyl-CoA hydrolase	Acetyl-CoA hydrolase	8-Amino-7-oxononanoate synthase
Aconitate hydratase (mitochondrial)	Acetyl-CoA synthase	**Aldehyde dehydrogenase**
Alcohol dehydrogenase	Aconitate hydratase (mitochondrial)	α,α-Trehalase
**Aldehyde dehydrogenase**	Adenylate kinase	α-Glucosidase
α-Glucosidase	Alanine-glyoxylate transaminase	α-Glucosidase
α-Glucosidase	Alcohol dehydrogenase	Aspartate-carbamoyltransferase
α-Glucosidase	**Aldehyde dehydrogenase**	Aspartate-carbamoyltransferase
Aspartate transaminase (mitochondrial)	Aspartate transaminase (mitochondrial)	B-ketoacyl-ACP synthase
Aspartate transaminase (mitochondrial)	Aspartate transaminase (mitochondrial)	Carbamoyl-phophate synthetase
ATP:citrate oxaloacetate-lyase	ATP:citrate oxaloacetate-lyase	**Citrate synthase (mitochondrial)**
Carnitine *O*-acetyltransferase	Carnitine *O*-acetyltransferase	Dihydrolipoamide *S*-acetyltransferase (mitochondrial)
Carnitine *O*-acetyltransferase (mitochondrial)	Carnitine *O*-acetyltransferase (mitochondrial)	Dihydroxy acid dehydratase (mitochondrial)
Carnitine/acyl carnitine carrier	**Citrate synthase (mitochondrial)**	Fatty-acyl-CoA synthase
**Citrate synthase (mitochondrial)**	**Citrate synthase (mitochondrial)**	Fatty-acyl-CoA synthase
Formate dehydrogenase	Formate dehydrogenase	Fructose-bisphosphatase
Fructose-bisphosphatase	Fumarate dehydratase (mitochondrial)	Glucan 1,3-β-glucosidase (extracellular)
Gluconolactonase (extracellular)	**Glucose 6-phosphate 1-dehydrogenase**	**Glucose 6-phosphate 1-dehydrogenase**
**Glucose 6-phosphate 1-dehydrogenase**	Glucose-6-phosphate isomerase	Glycerol 3-phosphate dehydrogenase (FAD dependent)
Glyceraldehyde 3-phosphate dehydrogenase	Glycerol 3-phosphate dehydrogenase (FAD dependent)	Glycerol dehydrogenase
Isocitrate lyase (glyoxysomal)	Glycerol dehydrogenase	Glycerol kinase
Glycerol kinase	Isocitrate lyase (glyoxysomal)	GTP cyclohydrolase I
Mannose-6-phosphate isomerase	Malate dehydrogenase (malic enzyme; NADP+)	Ketol-acid reductoisomerase (mitochondrial)
**Phosphoenolpyruvate carboxykinase**	Malate synthase (glyoxysomal)	Malate dehydrogenase (malic enzyme; NADP+)
**Pyruvate kinase**	Mannitol 2-dehydrogenase (NAD+)	Mannitol 2-dehydrogenase (NAD+)
Transketolase	**Phosphoenolpyruvate carboxykinase**	Mannitol 2-dehydrogenase (NADP+)
	Phosphoglucomutase	**Phosphoenolpyruvate carboxykinase**
	Phosphogluconate dehydrogenase (decarboxylating)	Phosphoribosylamine-glycine ligase
	Phosphorylase	Phosphorylase
	**Pyruvate kinase**	Pyruvate dehydrogenase (lipoamide) (mitochondrial)
	Transketolase	**Pyruvate kinase**
	UTP-glucose-1-phosphate uridylyltransferase	Ribulokinase
		UTP-glucose-1-phosphate uridylyltransferase

Shown is a list of the enzymes and transporters that participate in the 'small', highly correlated subnetworks for each pair-wise comparison between the three carbon sources investigated. Enzymes common to all reactions are highlighted in bold. Some enzymes appear more than once in the table, which means that they are isoenzymes and are encoded by different genes. CoA, coenzyme A.
