# Supplementary figures and images for: Metabolic network driven analysis of genome-wide transcription data from Aspergillus nidulans
Source: Genome Biol. 2006 Nov 15;7(11):R108. doi: 10.1186/gb-2006-7-11-r108 (PMC1794588; doi:10.1186/gb-2006-7-11-r108)

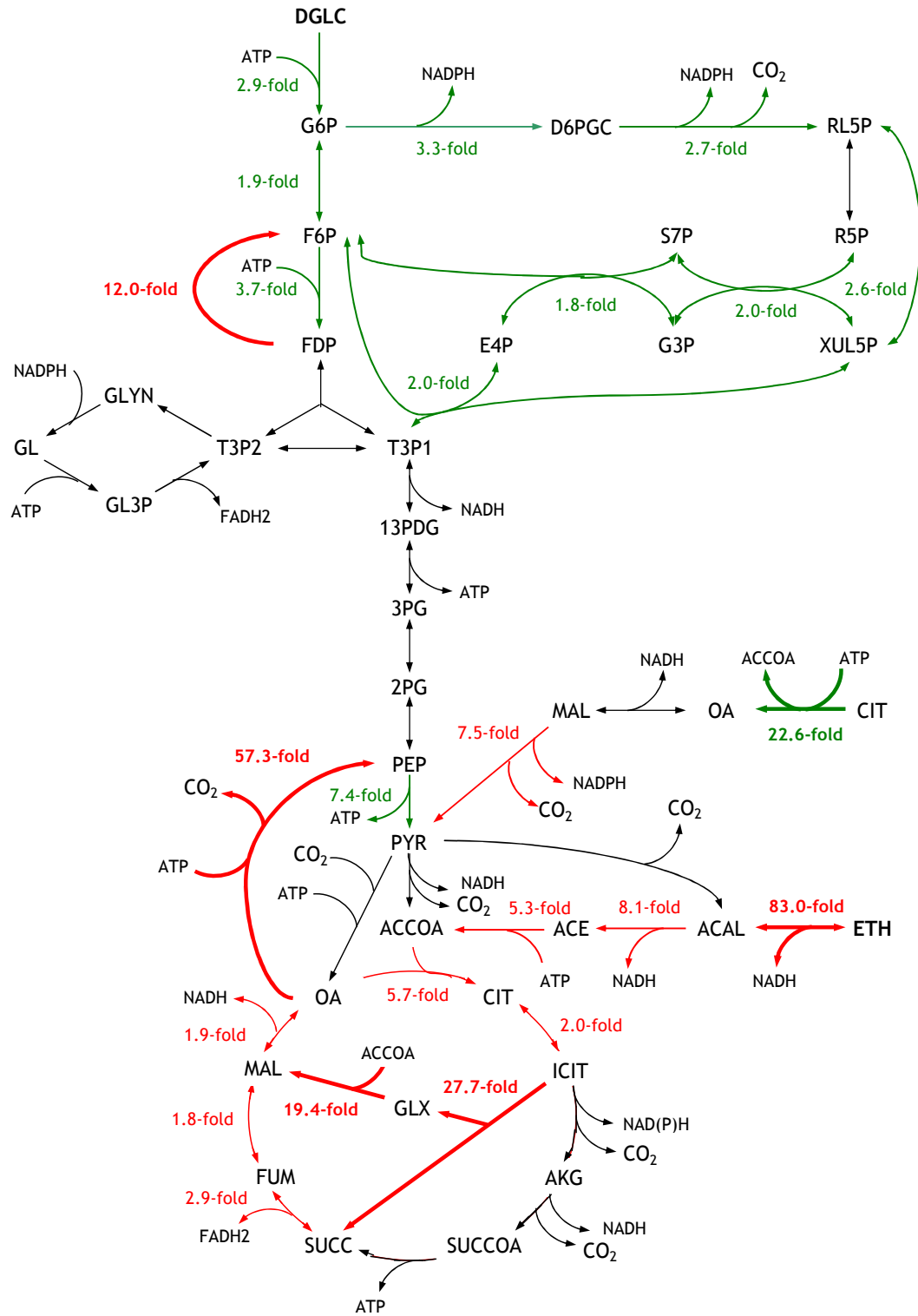

Supplement: Additional data file 1 — Differentially expressed genes in the central metabolism of A. nidulans between the replicated experiments on glucose and ethanol, revealed by the logit-t method (see Additional data file 9 [Table S6] for a full list of genes. Upregulated and downregulated genes are represented in red and green, respectively. Fold changes greater than 10 are highlighted in bold. [file gb-2006-7-11-r108-S1.pdf]

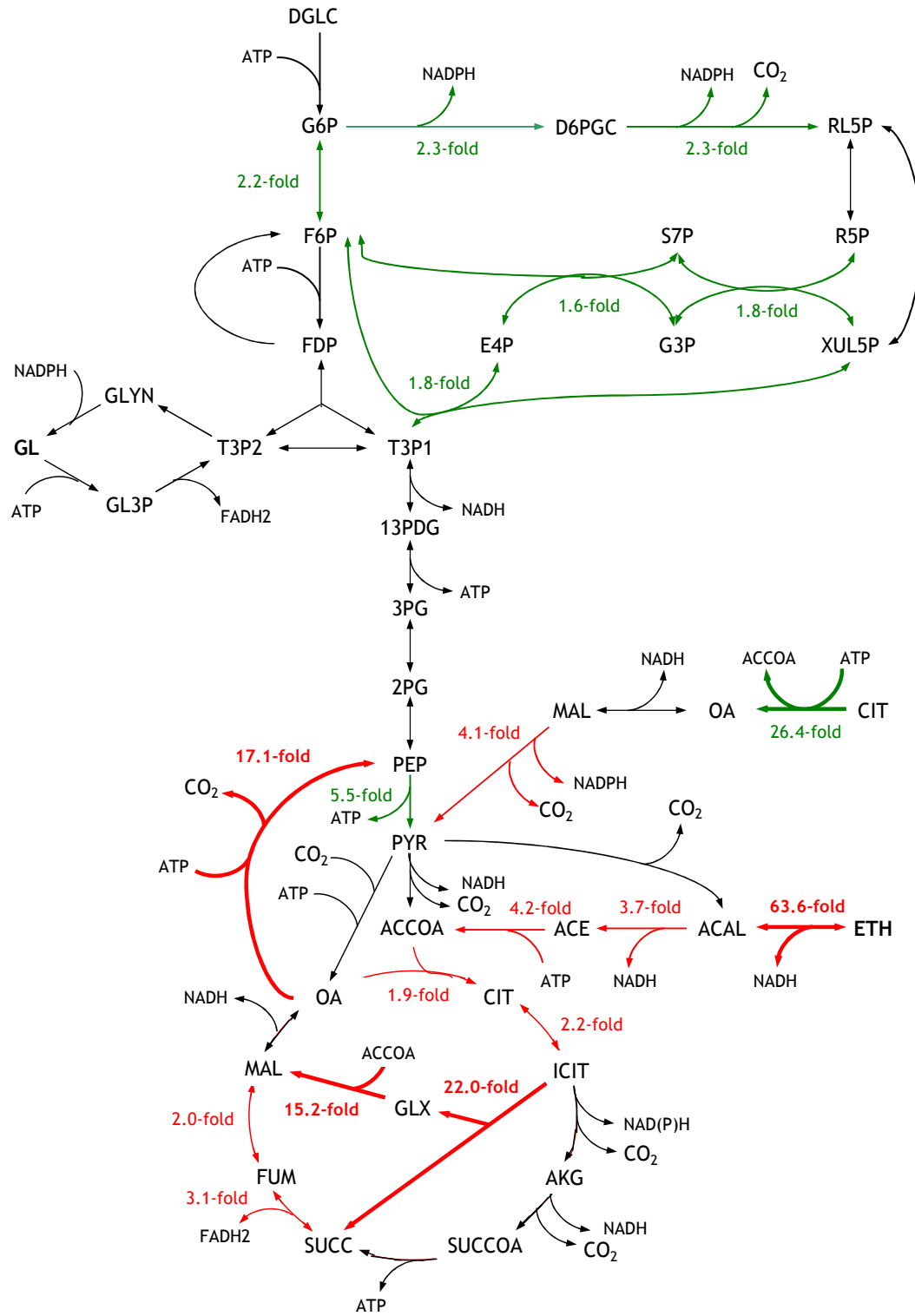

Supplement: Additional data file 2 — Differentially expressed genes in the central metabolism of A. nidulans between the replicated experiments on glycerol and ethanol, revealed by the logit-t method (see Additional data file 9 [Table S7] for a full list of genes. Upregulated and downregulated genes are represented in red and green, respectively. Fold changes greater than 10 are highlighted in bold. [file gb-2006-7-11-r108-S2.pdf]

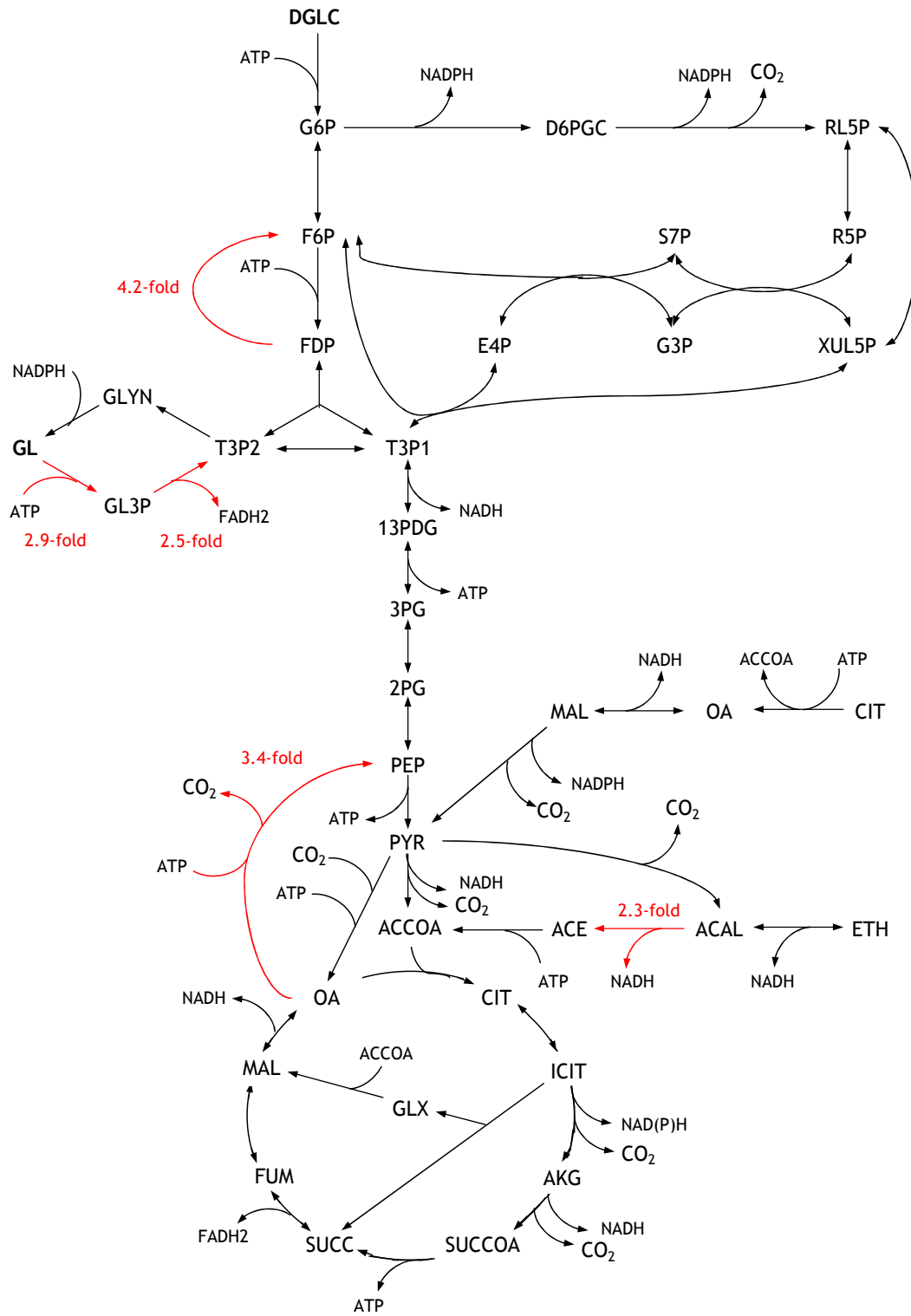

Supplement: Additional data file 3 — Differentially expressed genes in the central metabolism of A. nidulans between the replicated experiments on glucose and glycerol, revealed by the logit-t method (see Additional data file 9 [Table S8] for a full list of genes. Upregulated and downregulated genes are represented in red and green, respectively. Fold changes greater than 10 are highlighted in bold. [file gb-2006-7-11-r108-S3.pdf]

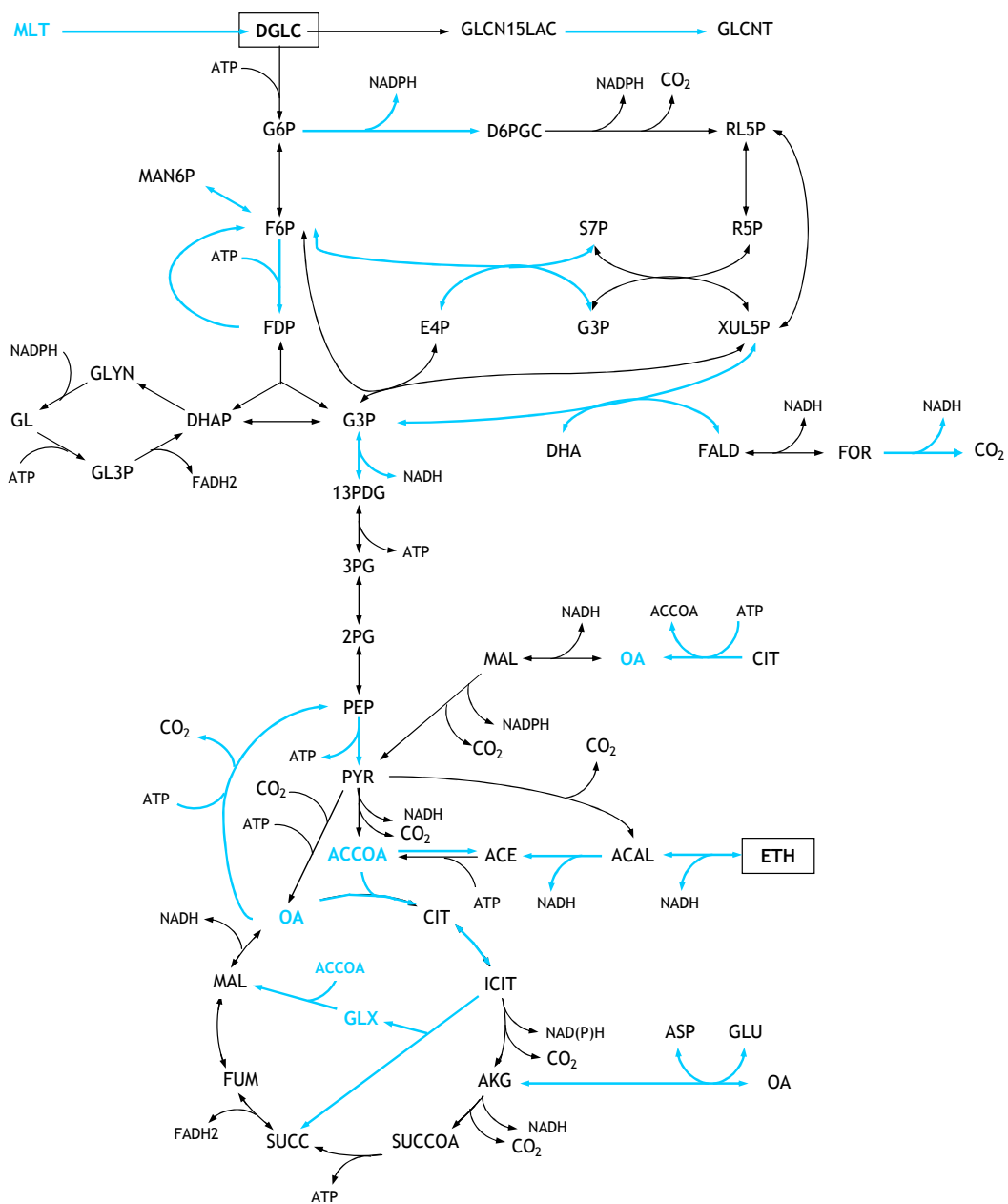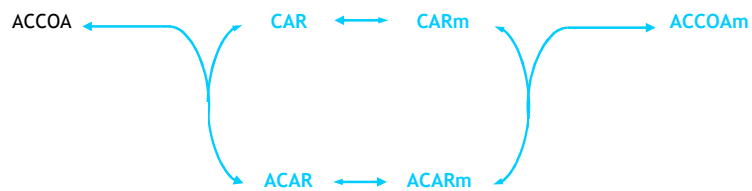

Supplement: Additional data file 4 — Reporter metabolites and enzymes comprising the 'small' subnetwork identified by comparing expression data on glucose and ethanol (represented in blue). Also shown are the top 15 high-scoring reporter metabolites. [file gb-2006-7-11-r108-S4.pdf]

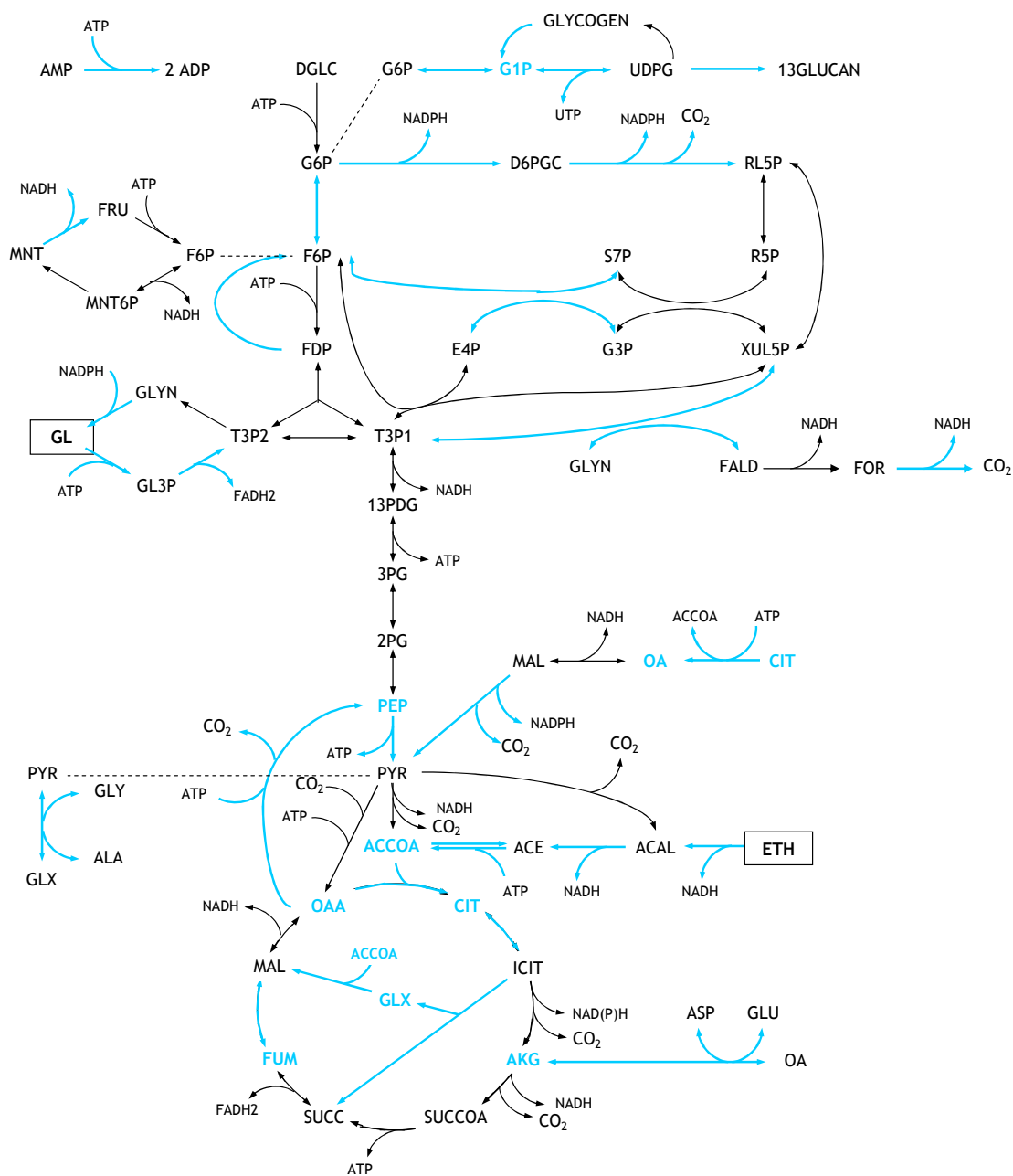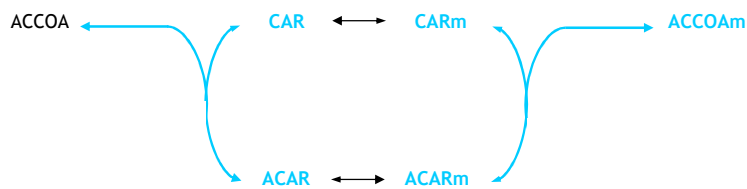

Supplement: Additional data file 5 — Reporter metabolites and enzymes comprising the 'small' subnetwork identified by comparing expression data on glycerol and ethanol (represented in blue). Also shown are the top 15 high-scoring reporter metabolites. [file gb-2006-7-11-r108-S5.pdf]

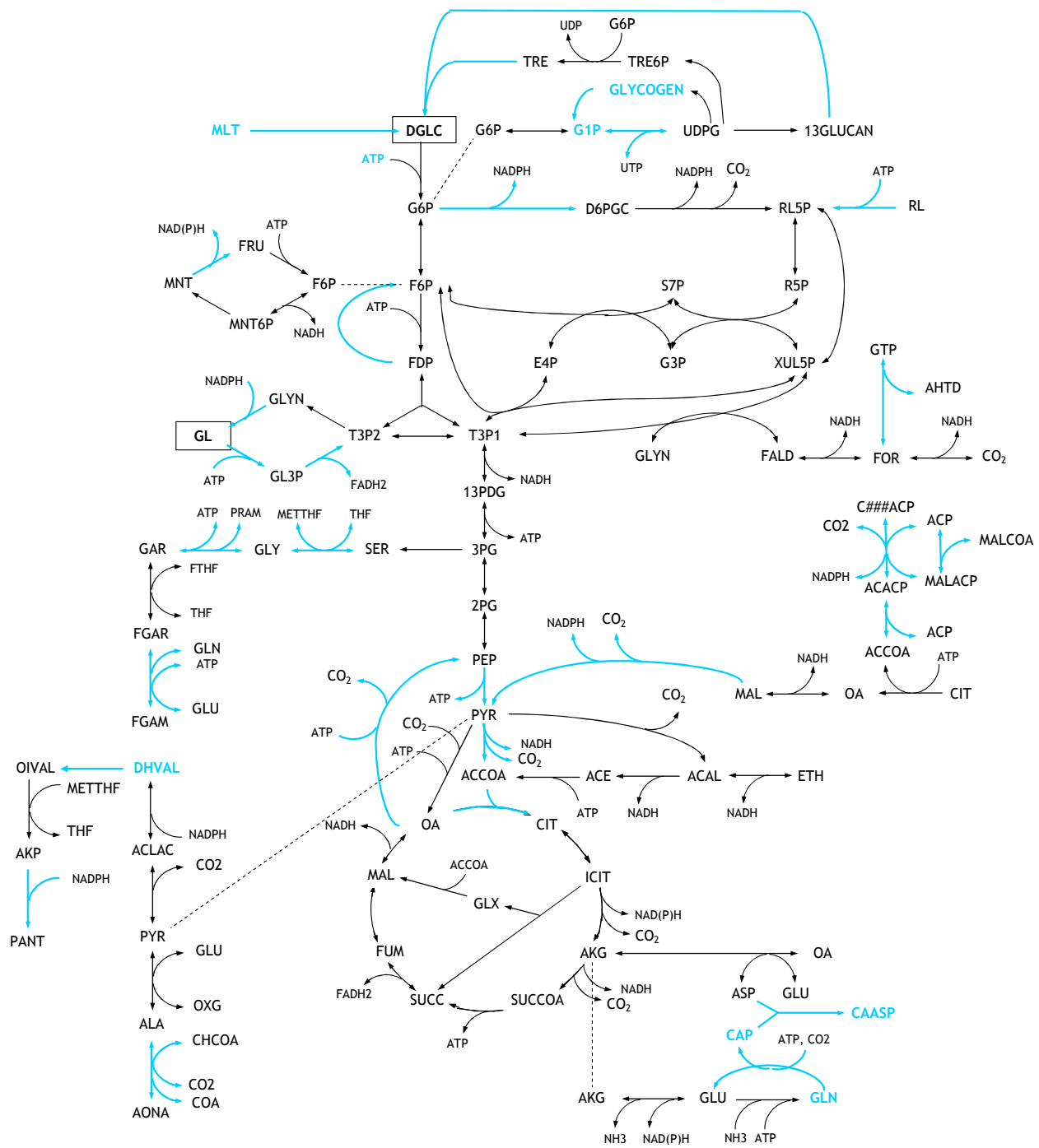

Supplement: Additional data file 6 — Reporter metabolites and enzymes comprising the 'small' subnetwork identified by comparing expression data on glucose and glycerol (represented in blue). Also shown are the top 15 high-scoring reporter metabolites. [file gb-2006-7-11-r108-S6.pdf]
