# Supplementary material for: Metabolic network driven analysis of genome-wide transcription data from Aspergillus nidulans
Source: Genome Biol. 2006 Nov 15;7(11):R108. doi: 10.1186/gb-2006-7-11-r108 (PMC1794588; doi:10.1186/gb-2006-7-11-r108)
Supplement: Additional data file 10 — Tables S9 and S10 present the classifications of the upregulated and downregulated genes, respectively, given in Table 2 into GO categories (provided by CADRE), according to the three most important biological processes and molecular functions. [file gb-2006-7-11-r108-S10.pdf]

Table S9 – Classification of the up-regulated genes represented in Table II into GO categories (provided by CADRE), according to the three most important biological processes and molecular functions.

	Biological processes	Molecular functions
**Ethanol vs Glucose**	• Generation of precursor metabolites and energy<br>• Main pathways of carbohydrate metabolism<br>• Acetyl-CoA metabolism	• Oxidoreductase activity<br>• Oxidoreductase activity, acting on the aldehyde or oxo group of donors<br>• Oxidoreductase activity, acting on the aldehyde or oxo group of donors, NAD or NADP as acceptor
**Ethanol vs Glycerol**	• Main pathways of carbohydrate metabolism<br>• Energy derivation by oxidation of organic compounds<br>• Organic acid metabolism	• Oxidoreductase activity<br>• Transaminase activity<br>• Transferase activity, transferring nitrogenous groups
**Glycerol vs Glucose**	• Cell wall<br>• Cell wall (sensu Fungi)<br>• External encapsulating structure	• Glucan 1,3-beta-glucosidase activity<br>• Beta-glucosidase activity<br>• Hydrolase activity, hydrolyzing O-glycosyl compounds

Table S10 – Classification of the down-regulated genes represented in Table II into GO categories (provided by CADRE), according to the three most important biological processes and molecular functions.

	Biological processes	Molecular functions
**Ethanol vs Glucose**	• Monosaccharide metabolism<br>• Hexose metabolism<br>• Alcohol metabolism	• Carbohydrate kinase activity<br>• Intramolecular oxidoreductase activity<br>• Isomerase activity
**Ethanol vs Glycerol**	• Glucose metabolism<br>• Hexose metabolism<br>• Carbohydrate metabolism	• Intramolecular oxidoreductase activity<br>• Hydrolase activity, hydrolyzing O-glycosyl compounds<br>• Isomerase activity
**Glycerol vs Glucose**	• Cell wall biosynthesis (sensu Fungi)<br>• Cell wall organization and biogenesis (sensu Fungi)<br>• Cell wall biosynthesis	• Heat shock protein activity<br>• Alpha-glucosidase activity<br>• Glucosidase activity
