# Supplementary material for: Metabolic network driven analysis of genome-wide transcription data from Aspergillus nidulans
Source: Genome Biol. 2006 Nov 15;7(11):R108. doi: 10.1186/gb-2006-7-11-r108 (PMC1794588; doi:10.1186/gb-2006-7-11-r108)
Supplement: Additional data file 14 — Table S17 summarizes the classification of the genes included in the 'small' highly correlated subnetworks into GO categories (provided by CADRE), according to the three most important biological processes and molecular functions. [file gb-2006-7-11-r108-S14.pdf]

Table S17 – Classification of the genes included in the "small" highly correlated subnetworks into GO categories (provided by CADRE), according to the three most important biological processes and molecular functions.

Subnetwork	No. of genes	Biological processes	Molecular functions
**Ethanol vs Glucose**	26	• Main pathways of carbohydrate metabolism<br>• Energy derivation by oxidation of organic compounds<br>• Cellular carbohydrate metabolism	• Catalytic activity<br>• Alpha-glucosidase activity<br>• Carnitine O-acetyltransferase activity
**Ethanol vs Glycerol**	33	• Carbohydrate metabolism<br>• Cellular carbohydrate metabolism<br>• Main pathways of carbohydrate metabolism	• Catalytic activity<br>• Transferase activity<br>• Transferase activity, transferring acyl groups, acyl groups converted into alkyl on transfer
**Glycerol vs Glucose**	34	• Pyruvate metabolism<br>• Organic acid metabolism<br>• Carboxylic acid metabolism	• Catalytic activity<br>• Carbon-nitrogen ligase activity, with glutamine as amido-N-donor<br>• Carbamoyl-phosphate synthase activity
